# Preventable deaths involving opioids in England and Wales, 2013–2022: a systematic case series of coroners’ reports

**DOI:** 10.1093/pubmed/fdad147

**Published:** 2023-08-21

**Authors:** Francesco Dernie, Harrison S France, Elizabeth T Thomas, Maja Bilip, Nicholas J DeVito, Robin E Ferner, Anthony R Cox, Carl Heneghan, Jeffrey K Aronson, Georgia C Richards

**Affiliations:** Oxford University Hospitals NHS Trust, Oxford OX3 9DU, UK; Oxford Medical School, Medical Sciences Divisional Office, University of Oxford, Oxford OX3 9DU, UK; Centre for Evidence-Based Medicine, Nuffield Department of Primary Care Health Sciences, University of Oxford, Radcliffe Observatory Quarter, Woodstock Road, Oxford OX2 6GG, UK; Department of Internal Medicine, Thames Hospital, MacKay Street, Thames 3500, New Zealand; Bennett Institute for Applied Data Science, Nuffield Department of Primary Care Health Sciences, University of Oxford, Radcliffe Observatory Quarter, Woodstock Road, Oxford OX2 6GG, UK; Institute of Clinical Sciences, University of Birmingham, Birmingham, UK; West Midlands Centre for Adverse Drug Reactions, City Hospital, Birmingham B17 7QH, UK; Institute of Clinical Sciences, University of Birmingham, Birmingham, UK; West Midlands Centre for Adverse Drug Reactions, City Hospital, Birmingham B17 7QH, UK; Centre for Evidence-Based Medicine, Nuffield Department of Primary Care Health Sciences, University of Oxford, Radcliffe Observatory Quarter, Woodstock Road, Oxford OX2 6GG, UK; Centre for Evidence-Based Medicine, Nuffield Department of Primary Care Health Sciences, University of Oxford, Radcliffe Observatory Quarter, Woodstock Road, Oxford OX2 6GG, UK; Centre for Evidence-Based Medicine, Nuffield Department of Primary Care Health Sciences, University of Oxford, Radcliffe Observatory Quarter, Woodstock Road, Oxford OX2 6GG, UK

**Keywords:** addiction, drug abuse, mortality, opioid

## Abstract

**Background:**

Opioid deaths have increased in England and Wales. Coroners’ Prevention of Future Deaths reports (PFDs) provide important insights that may enable safer use and avert harms, yet reports implicating opioids have not been synthesized. We aimed to identify opioid-related PFDs and explore coroners’ concerns to prevent future deaths.

**Methods:**

In this systematic case series, we screened 3897 coronial PFDs dated between 01 July 2013 and 23 February 2022, obtained by web scraping the UK’s Courts and Tribunals Judiciary website. PFDs were included when an opioid was implicated in the death. Included PFDs were descriptively analysed, and content analysis was used to assess concerns reported by coroners.

**Results:**

Opioids were involved in 219 deaths reported in PFDs (5·6% of PFDs), equating to 4418 years of life lost (median 33 years/person). Morphine (29%), methadone (23%) and diamorphine (16%) were the most common implicated opioids. Coroners most frequently raised concerns regarding systems and protocols (52%) or safety issues (15%). These concerns were most often addressed to National Health Service (NHS) organizations (51%), but response rates were low overall (47%).

**Conclusions:**

Opioids could be used more safely if coroners’ concerns in PFDs were addressed by national organizations such as NHS bodies, government agencies and policymakers, as well as individual prescribing clinicians.

## Introduction

In 2019, the UK had the highest rate per capita of opioid consumption globally.^1^ Prescriptions of opioids in primary care and the rates of opioid-related hospitalization in the UK have both increased contemporaneously.^2–4^ In England and Wales, deaths from opioids have increased nearly 5-fold in under three decades, from 8·4 per million of the population in 1993 to 39·7 per million of the population in 2020.^5^ There are calls for the UK Government to recognize the rise in opioid-related deaths as a public health crisis.^6^^,^^7^

In England and Wales, unnatural or unclear causes of deaths are established at inquests conducted by coroners. Since 1984, coroners have had a duty to report and communicate a death when they believe that action can be taken to prevent future deaths.^8^ These reports, named Prevention of Future Deaths reports (PFDs) are now mandated by the Coroners and Justice Act 2009 and the Coroners (Investigations) Regulations 2013.^9^^,^^10^

Studies have systematically analysed PFDs to assess deaths during the COVID-19 pandemic, involving anticoagulants, medicines purchased online, medication errors and adverse drug reactions.^11–15^ A case series of medicine-related PFDs showed that opioids were the most common drug involved in deaths.^16^ However, these concerns involving opioids have not been synthesized. A systematic assessment of opioid-related PFDs and their associated responses might therefore provide valuable information to inform public health strategies to reduce opioid deaths.

The aim of our study was to conduct a systematically case series of PFDs to identify and characterize deaths involving opioids, synthesize coroners concerns and classify the responses of individuals or organizations to whom PFDs were addressed.

## Methods

A systematic case series was designed and the study protocol was preregistered on an open repository.^17^

### Data collection

Data were acquired from the Courts and Tribunals Judiciary website^18^ using web scraping^19^ to populate the Preventable Deaths Database,^20^ which included 3897 PFDs as of 23 February 2022. The code that generates the database is openly available.^21^

### Data screening and eligibility

The 3897 PFDs were independently screened by study authors (GCR, MB, HF, FD) using a predefined definition for opioids; any substance, either natural, synthetic or semi-synthetic, ‘derived from or having properties similar to those of morphine’.^22^ Cases were included if an opioid was believed to have caused or contributed to the death, including licit and illicit opioids, as per a pre-defined algorithm for case inclusion (Supplementary Fig. S1).

### Data extraction

The web scraper automatically extracted the case reference number; date of the PFD report; name of the deceased; coroner’s name; coroner’s area; category of death (as defined by the Chief Coroner’s Office); the institution(s) or individual(s) to whom the report was sent; and the URL of the case.

The investigators (FD, GCR, HSF, ETT) manually extracted the following variables into the database: the dates on which the inquest began and ended; number of deceased mentioned in the PFD; dates of death(s); sex; age; setting/location of death; any relevant medical and/or social history; medical cause(s) of death and/or conclusion of the coroner’s inquest; substance(s) implicated in deaths, including any reported opioids; details of the coroner’s concern(s); the number and types of individual(s) or organization(s) to whom the reports were sent; whether the individuals or organizations addressed responded (yes/no), the date of the response, and the number of days under or overdue.

We also extracted mortality statistics for England and Wales on opioid-related deaths from the Office for National Statistics (ONS) from 2013 to the most up-to-date data (2020)^5^ at time of analysis (April 2022) to show the rates of opioid-related deaths that had a PFD written.

### Data analysis

The number of included opioid-related PFDs, their rates as a proportion of all PFDs, and ONS mortality data were plotted over time. Medians and interquartile ranges (IQRs) were calculated for continuous variables (e.g. age) and frequencies were reported for categorical variables (e.g. sex, type and source of opioid, coroner area of jurisdiction).

We calculated the years of life lost (YLL) for the deceased with available ages using the formula, $YLL=\sum \left(E-A\right);$where *E* = life expectancy (World Health Organization estimate of 75 years); and *A* = age of subject at death.^23^ When age at death was over 75, these were described individually and counted as ‘0’ YLL.

One investigator (FD) assigned the International Statistical Classification of Diseases and Related Health Problems 10^th^ Revision (ICD-10)^24^ numeric codes for the causes of death to each PFD and compared the number of PFDs correctly categorized under the alcohol, drug and medication related deaths section on the Judiciary website.

To examine geographical variation, we graphed both the absolute counts and rates of opioid-related PFDs per all PFDs in each coroner area mapped to the standard regions of England and Wales.

To collate and evaluate concerns raised by coroners, we classified and identified repeated themes using directed content analysis.^25^ This allowed us to highlight cases involving previously recognized concerns, and to explore concepts, drawing similarities and disparities from the data.

To calculate response rates to PFDs, we used the 56-day legal requirement^9^ to classify responses as ‘early’ (>7 days before due date), ‘on-time’ (±7 days before due date), ‘late’ (>7 days after due date) or ‘overdue’ (response was not available on the Judiciary website as of 23 February 2022). We calculated the average response rate and frequency for individuals and organizations.

Available demographics and coroners’ concerns were also reported by the source of opioids (i.e. prescribed versus illicit opioids) in a sub group analysis.

### Software and data sharing

We used Datawrapper^26^ to produce a choropleth map of England and Wales, and Tableau^27^ software to generate our bubble chart of coroners’ concerns. The study protocol, materials and statistical code are openly available via the Open Science Framework^17^^,^^28^ and GitHub.^21^

## Results

**Fig. 1:**
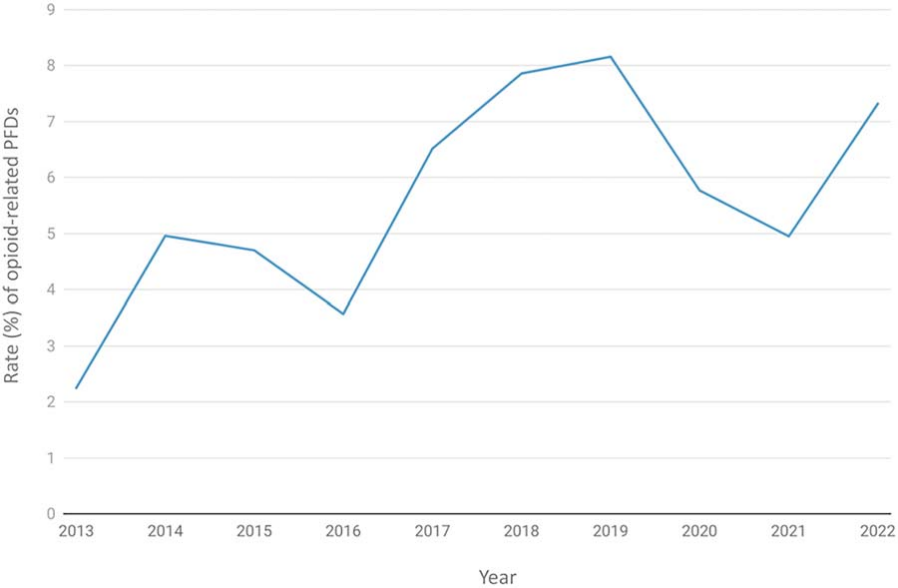
The rate of opioid-related Prevention of Future Deaths reports (PFDs) as a proportion of all PFDs (*n* = 3897) published overtime, between 31 July 2013 and 23 February 2022 in England and Wales.

There were 219 opioid-related PFDs and deaths between July 2013 and 23 February 2022 in England and Wales (5·6% of all PFDs). The rate of opioid-related PFDs increased by 217% from 2.3% in 2013 to 7·3% in 2022 (Fig. 1). Compared with ONS statistics, a median of 1·2% (IQR: 0·84–1·29) of all opioid-related deaths in England and Wales were written into PFDs each year (Supplementary Table S1).

In 137 of these preventable deaths reporting ages under 75 years, 4418 years of life were lost, a median of 33 years per person (IQR: 24–42·5; *n* = 137). The median age at death was 42 years (IQR: 32·5–51 years; *n* = 137) and five individuals were older than 75 years at the time of death. The average age at death was almost a decade younger in those that sourced illicit opioids (35 years; IQR: 30·3–44; *n* = 32) compared with prescribed opioids (43 years; IQR: 31–58·3; *n* = 70 Supplementary Table S2).

In all opioid-related PFDs, most (64%, *n* = 141) of those who died were male. However, those that obtained illicit opioids had a higher proportion of males (86·5%; *n* = 52) compared to the prescribed group (50%; *n* = 144; Supplementary Table S2).

On the Judiciary website, only 37% (*n* = 82) of opioid-related PFDs were classified under the ‘Alcohol, drug and medication related deaths’ report type and 4% (*n* = 8) were classified as ‘Product related deaths’ (Supplementary Table S3). However, when applying the ICD-10 categorization, the most common (41%) causes of death were poisoning by ‘other’ opioids (e.g. morphine, codeine), followed by poisoning by methadone (20%) and poisoning by other synthetic narcotics (19%) (Supplementary Table S4).

**Fig. 2:**
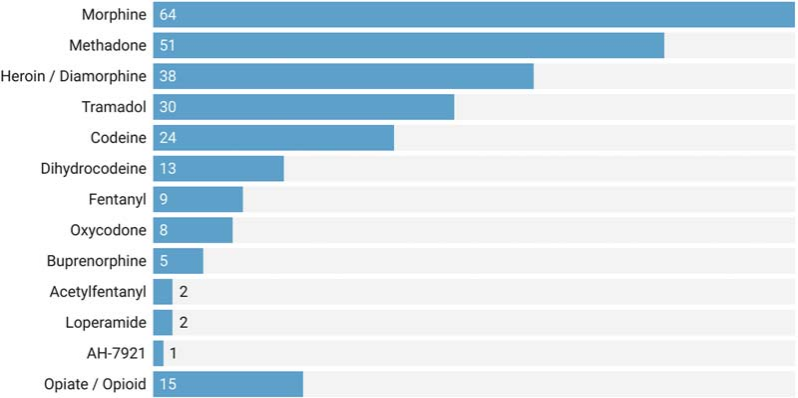
Rates of opioid drugs reported in 219 Prevent Future Death (PFD) reports published between July 2013 and February 2022 in England and Wales. Some PFDs reported multiple opioids and others did not specify the type of opioid involved (i.e. the opiate/opioid category).

Morphine (29%) was the most frequently implicated opioid in deaths, followed by methadone (23%) and heroin (16%) (Fig. 2). Most (52%, *n* = 114) of the deaths involved opioids that were prescribed, followed by illicit drugs (24%, *n* = 52), and a combination of licit and illicit (14%, *n* = 31). In 22 cases (10%) the source of the drug was unclear or unstated. Those for whom opioids were prescribed commonly received analgesics and methadone, compared with those who obtained illicit opioids, who most often sourced heroin (Supplementary Fig. S2).

**Fig. 3:**
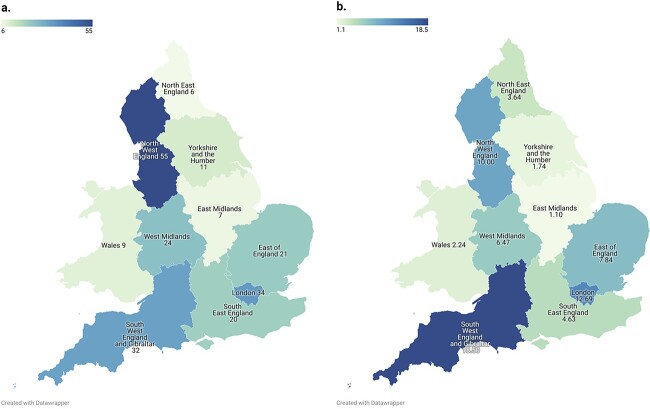
Choropleth map of the regions of England and Wales from which the 219 opioid-related Prevention of Future Deaths reported (PFDs) were issued by coroners between July 2013 and February 2022, showing the raw numbers of PFDs (**a**), and the rate (%) of opioid-related PFDs per all PFDs (**b**).

The 219 PFDs were written by 125 coroners across 71 jurisdictions (Supplementary Table S5). Coroners in the North-West of England wrote the most (25%, *n* = 55/219) opioid-related PFDs (Fig. 3a); however, after correcting for the number of PFDs written in each area, the highest rate of opioid-related PFDs (18·5%) was reported in the South-West of England (Fig. 3b).

**Fig. 4:**
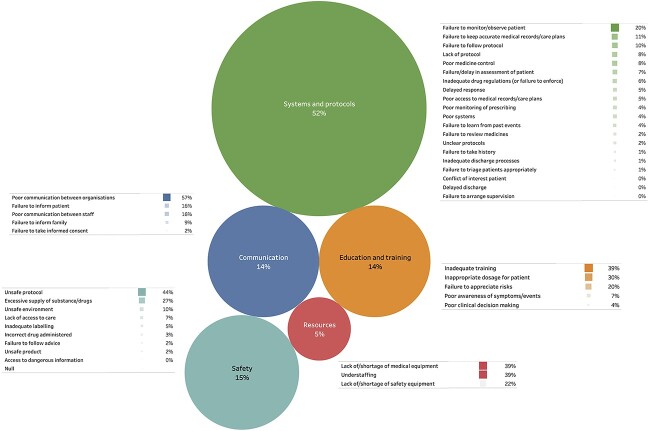
Concerns raised by coroners in 219 opioid-related Prevention of Future Deaths reports (PFDs) in England and Wales published between July 2013 and February 2022. The size of each circle is representative of the frequency of the five higher-order themes. The individual concerns and their frequencies are reported in adjacent lists.

Coroners expressed 666 individual concerns in the 219 opioid-related PFDs. Using content analysis, these concerns were categorized into 42 separate themes with five higher-order themes, which were systems and protocols, communication, safety, education and training, and resources. In all opioid-related PFDs, concerns were most often related to systems and protocols (52%, Supplementary Table S6), and this was reflected in both the illicit (51%) and prescribed (51%) groups (Supplementary Table S7). The most common individual concerns for all opioid-related PFDs were the failure to monitor/observe patients (10%), poor communication between organizations (8%) and unsafe protocols (7%; Fig. 4; Supplementary Table S6). In people for whom opioids were prescribed, concerns of excessive supply and inappropriate dosing were more common than in those who used illicit opioids (Supplementary Table S7).

Coroners sent 360 PFDs to 43 unique organizations (Supplementary Table S8). The National Health Service (NHS) in England and Wales received the most (51%) PFDs; however most (53%) of their responses were overdue (i.e. no response within 56 days of receiving the report). The response rate varied widely between organizations: 36% responded early or on time, 11% responded late and 53% remained overdue as of 23 February 2022.

## Discussion

### Main findings of this study

There were 219 deaths involving opioids where coroners believed actions should be taken to prevent similar deaths. On average, 32 years of life were lost for every opioid-related PFD. Morphine, methadone and heroin were responsible for most deaths, which frequently occurred in London, the North-West and the South-West of England. Coroners raised repeated concerns relating to systems and protocols, such as failures to arrange appropriate monitoring or follow-up, and the maintenance of accurate medical records and plans of patients receiving opioids. These concerns were sent to hundreds of organizations, mostly healthcare providers and NHS services. Despite the legal requirements to do so, less than half of all recipients have responded, or have had their responses published in the public domain. It is therefore unclear what actions are being taken to prevent avoidable opioid-related deaths.

### What is already known on this topic

Several studies have investigated coroners’ concerns in PFDs.^11–16^ These studies have similarly found poor response rates from recipients receiving PFDs^11^^,^^29^ and wide geographical variation in the writing of reports,^11^^,^^30^ concerns that were also highlighted in a 2021 report by the House of Commons Justice Select Committee.^31^ A case series of anticoagulant-related PFDs similarly showed that systems and protocols were the most frequent coroners’ concerns.^11^

Several of our findings relating to opioid-related deaths have been reflected in the literature. An observational study of 76 countries published in 2021 showed that the UK had the highest rate of opioid consumption, which was driven by a greater use of tramadol and codeine in the UK^1^ compared with other countries. In line with this research, we found that tramadol and codeine were the fourth and fifth most common opioids implicated in deaths. An analysis of over-the-counter sales of codeine in 31 countries also showed that the UK had the fourth highest rates of sales,^32^ which may have contributed to the one in ten codeine-related PFD deaths we identified. A retrospective cohort study of adults in Glasgow, Scotland, showed that the co-occurrence of opioid dependence with homelessness, involvement with criminal justice services, and psychosis was associated with higher rates of premature mortality.^33^ Similarly, we found that 14% of the PFDs in our analysis were categorized on the Courts and Tribunals Judiciary website as ‘Mental health related deaths’ and 13% as ‘State Custody’ and ‘Police related deaths’. A matched cohort analysis of causes of deaths of English people who used illicit opioids showed that fatal poisonings were more common among younger individuals, whereas older individuals were more likely to die from non-communicable diseases.^34^ This may explain the lower average age of death among those who sourced opioids illicitly in PFDs. A case cross-over study of opioid-related deaths showed that while the prescription of high-dose opioids or co-prescription of gabapentinoids or antidepressants increased the risk of death, considerable proportions of individuals had not been given an opioid in the year prior to death (26·7%) or had been given relatively low doses (22%).^35^ This suggests that a considerable number of deaths may have been accidental overdoses, as a result of illicit use or use of stockpiled prescribed drugs, both of which are scenarios found in several of the cases in our analysis.

### What this study adds

Several studies have investigated coroners’ concerns in PFDs,^11–16^ yet none has focused exclusively on opioid-related deaths, even though opioids have previously been implicated as the most common medication type causing preventable deaths.^16^ We efficiently collected all available PFDs for this study using reproducible methods, which are openly available.^19^

Understanding causes of death and how they can be prevented is critical for improving health, economic and social outcomes globally. Both Public Health England (PHE) and the British Medical Association have released reports that identified the widespread use and harms of opioids with recommendations to tackle these issues,^36^^,^^37^ but these recommendations have largely remained unimplemented.^36^^,^^37^ The UK Government published a 10-year drugs strategy in 2021, but concerns have been raised about its focus and its potential efficacy in the long-term.^38^ Our findings, including sub-group differences and content analysis of concerns, add to these reports by providing insight into the causes of deaths involving opioids that can be prevented. NHS England report that they review PFDs to develop guidance and advice;^39^ however, it is not clear how the insights from PFDs are currently being used in practice and policy. Our report aims to disseminate findings from these reports more widely.

### Limitations of this study

The 219 opioid-related PFDs identified cannot represent all preventable deaths involving opioids in England and Wales as this represents only a small percentage of all opioid-related deaths, and coronial variation in the writing of PFDs has been reported.^31^^,^^40^ We controlled for geographical variation by reporting opioid-related PFDs as a proportion of all PFDs and the rates for each geographical region. However, the issuing of PFDs depends on the working practices of individual coroners, who, although required to submit PFDs when they consider that actions should be taken to prevent further deaths, are not subject to auditing or quality assurance of the reports they write. We also identified missing information in the PFDs; for example, 37% of PFDs did not include age at death or date of birth. This could be improved by standardizing the reporting of information in PFDs using electronic forms and providing PFD training for coroners, which will improve this important information source in the future.

In conclusion, we have used reproducible data-collection methods to comprehensively analyse all available PFDs, which provide valuable lessons for preventing future opioid-related deaths. The dissemination of these lessons remains limited, but implementation of their findings in future guidelines and clinical practice will improve patient safety and reduce the years of life lost from opioids-related deaths.

## Supplementary Material

Supplementary_material_21_12_22_fdad147

## Data Availability

All study materials, data and statistical code are openly available via online repositories. The study protocol was preregistered on the Open Science Framework (OSF; https://doi.org/10.17605/OSF.IO/QJE8A); the code to generate the database and the Preventable Deaths Tracker is openly available via GitHub (https://github.com/georgiarichards/georgiarichards.github.io); individual Prevention of Future Deaths reports are available on the Courts and Tribunals Judiciary website (https://www.judiciary.uk/prevention-of-future-death-reports/); all other study materials are openly available via the OSF project page (https://doi.org/10.17605/OSF.IO/ECZ4R).

## Notes

**Francesco Dernie,** Foundation Doctor

**Harrison S. France,** Medical Student

**Elizabeth T. Thomas,** Doctoral Researcher

**Maja Bilip,** House Doctor

**Nicholas J. DeVito,** Research Fellow

**Robin E. Ferner,** Professor

**Anthony R. Cox,** Professor

**Carl Heneghan,** Professor

**Jeffrey K. Aronson,** Clinical Pharmacologist

**Georgia C. Richards,** Research Fellow
